# Imported *Plasmodium vivax* malaria with severe thrombocytopaenia: can it be severe malaria or not?

**DOI:** 10.1186/s12936-016-1150-8

**Published:** 2016-02-19

**Authors:** Spinello Antinori, Alberto Corona, Anna Lisa Ridolfo, Laura Galimberti, Davide Ricaboni, Laura Milazzo, Mario Corbellino

**Affiliations:** Department of Clinical and Biomedical Sciences Luigi Sacco, III Division of Infectious Diseases, University of Milano, Via GB Grassi, 74, 20157 Milan, Italy; Intensive Care Unit Luigi Sacco Hospital, Milan, Italy

**Keywords:** *Plasmodium vivax*, Severe thrombocytopaenia, Severe malaria, Imported malaria, Bleeding

## Abstract

**Background:**

Thrombocytopaenia is the most frequent malaria-associated haematologic alteration observed with all five *Plasmodium* parasites causing disease in humans. Although not included in the World Health Organization criteria for severe *Plasmodium falciparum* malaria, severe thrombocytopaenia has been increasingly mentioned as an indicator of *P. vivax* malaria severity.

**Case:**

Here, it is described a case of imported *P. vivax* malaria in a 37-year old man from Pakistan who presented with severe thrombocytopaenia (5 × 10^9^/L). He was admitted to the intensive care unit and initially treated with a 1-day course of intravenous quinine followed by oral chloroquine and primaquine. The patient’s platelet count increased as early as 4 hours after treatment inception and the clinical course was favourable and uneventful.

**Discussion:**

This case report, along with a review of published cases focusing on the relationship between thrombocytopaenia and severe *P. vivax* malaria, suggests that the prognostic role of severe thrombocytopaenia is ambiguous in absence of severe haemorraghic complications and its use as diagnostic criterion of malaria severity may lead to overestimation of severe *P. vivax* malaria cases.

**Conclusion:**

Due to the lack of high quality studies it is at present unclear if severe thrombocytopaenia in the setting of *P. vivax* malaria should be considered indicative of severe malaria.

## Background

*Plasmodium vivax* is responsible for nearly half of all malaria cases diagnosed outside sub-Saharan Africa and it exhibits the widest geographical distribution of human malaria parasites with an estimated 2.49 billion individuals living in areas at risk of infection [1]. *Plasmodium vivax* malaria has been long known as “benign tertian malaria” as opposed to the “malignant tertian or sub-tertian malaria” caused by *P. falciparum* [2]. However, in recent years an increasing number of studies, especially from the Indian subcontinent and South America, have highlighted the role of *P. vivax* as a cause of severe and even fatal malaria [3–7]. For this reason, Baird recommended, in his revision of the nomenclature of human malaria, to include vivax malaria as an “acute pernicious” entity in terms of clinical presentation and evolution [6].

However, conflicting issues emerge from cases of severe *P. vivax* malaria that are increasingly reported in the literature, partly because of the lack of a clear definition of the severity criteria, and partly from the possible interference of concomitant morbidities and infections on the clinical presentation and outcome [8].

Low platelet counts are commonly encountered in all types of malaria, and values lower than 60,000/μL have been reported in 29–46 % of patients affected with vivax malaria [9, 10]. However, thrombocytopaenia is not regarded as a severity biomarker in falciparum malaria, and it has not as yet been validated as an independent severity parameter in vivax malaria. Of note, in several studies and case reports, severe thrombocytopaenia was described as the most prevalent severity sign of vivax malaria [11–15]. Therefore, it cannot be excluded that the use of such an indicator may lead to an overestimation of *P. vivax* malaria severity.

Here, it is described a case of imported *P. vivax* malaria characterized by severe thrombocytopaenia along with a review of similar cases reported in the literature. Furthermore, it is discussed whether severe thrombocytopaenia should be considered a reliable biomarker of *P. vivax* malaria severity.

## Case report

A 37-year old male from Pakistan arrived in Italy in July 2013 to attend a professional course. After few days, he developed high grade fever (40 °C) associated with chills, headache and one episode of vomiting. He was visited by a primary physician who prescribed him cefixime 400 mg/day and acetaminophen. After 4 days, due to the persistence of fever and the onset of productive cough and asthenia, cefixime was changed to levofloxacin (500 mg/day). However, the fever did not resolve, and therefore the patient was taken to the nearest emergency department (ED). At presentation, he was febrile (39.5 °C), tachycardic (121 beats per min) and hypotensive (90/50 mm Hg); there were no meningeal signs and the Glasgow Coma Score was normal (15). Oxygen saturation while breathing ambient air was 99 %, the respiratory examination and a chest radiograph were both normal. Laboratory investigations were as follows: haemoglobin, 15.9 g/dL; red blood cell count, 5.54 × 10^9^/L; total white cell count, 6.3 × 10^9^/L (with 85 % neutrophils, 11 % lymphocytes and 4 % monocytes); platelet count, 14 × 10^9^/L. With the exception of d-dimer concentration (9376 ng/dL), the other coagulation parameters were within the normal range. Hyponatremia (127 mmol/L) was present as well as raised values of C reactive protein (126 mg/L), total bilirubin (3.3 mg/dL), and lactate dehydrogenase (371 U/L). Serum concentrations of glucose, liver enzymes, urea and creatinine were within normal ranges. An ultrasound scan of the abdomen showed no hepatosplenomegaly and a normal biliary tract. *Legionella* and pneumococcus urine antigens were negative. A thin blood film showed “malaria parasites” that were not identified at the species level by the laboratory technician. Due to lack of anti-malarial drugs and the expertise for the diagnosis and therapy of malaria, the ED physician contacted the Luigi Sacco hospital in Milan for assistance. Ten hours after presentation at the first ED, the patient was finally admitted to the intensive care unit of Luigi Sacco hospital with a provisional diagnosis of “severe malaria”, based on the results of the laboratory tests (severe thrombocytopaenia and raised bilirubin levels) and the presence of hypotension. On physical examination, when admitted to the ICU, the patient was conscious, fully oriented to time and place with high fever (40 °C) and had severe headache. The pulmonary, cardiovascular and abdominal examinations were reportedly unremarkable. Neither signs of bleeding nor skin petechiae were detected. No previous history of malaria episodes was elicited and no underlying chronic diseases were present. The patient was treated with a loading dose of intravenous quinine (1200 mg). At treatment inception the platelet count was 5 × 10^9^/L, without haemorrhagic manifestations. Examination of the peripheral blood smear by the haematologist did not reveal platelet clumping and a blood sample obtained with sodium citrate confirmed the finding of low platelet counts thus excluding a diagnosis of pseudothrombocytopaenia. In the absence of bleeding the haematologist judged as unnecessary the use of platelet transfusions. Thick and thin blood smear examinations were diagnostic for *P. vivax* infection with schizonts, gametocytes and ring forms being detected and a very low parasitaemia (0.1 %). *Plasmodium vivax* monoinfection was subsequently confirmed by DNA polymerase chain reaction performed on a stored blood sample obtained on admission. Blood and urine cultures were obtained but gave negative results. Serology for HIV, CMV, dengue virus, leptospirosis, syphilis, *Brucella* and typhoid fever were negative.

Platelet counts showed a rapid increase soon after the infusion of quinine (i.e., 13 × 10^9^/L, 16 × 10^9^/L, 20 × 10^9^/L, 32 × 10^9^/L, respectively, after 4, 10, 18 and 24 h). The following morning an infectious diseases consultation was requested which suggested an interruption of intravenous quinine (after two doses administered). In consideration of the stable clinical condition and the diagnosis of *P. vivax* malaria the patient was transferred to the Infectious Diseases ward where oral chloroquine was started to complete the treatment. Parasite clearance was observed within 48 h after hospital admission as well as improvement or complete disappearance of all accompanying signs and symptoms (fever, headache, nausea, asthenia, myalgia). After completing a 3-day course of chloroquine treatment, and documentation of normal glucose 6-phosphate dehydrogenase activity levels, the patient was started on oral primaquine (15 mg of base in a 26.3 mg tablet per day for 14 days) and discharged the fifth day of hospitalization in good clinical conditions and with platelet counts within the normal range (223 × 10^9^/L).

## Discussion

In this case report, a man from Pakistan with imported *P. vivax* malaria was initially classified as affected by severe malaria and admitted to the ICU on the basis of profound thrombocytopaenia. Despite the initial presentation, the patient’s clinical course was uneventful and the platelet counts recovered very rapidly under anti-malarial treatment [16]. In recent years, an increasing number of case reports and hospital-based studies, have questioned the notion of “benign tertian malaria” by describing severe and even fatal malaria associated with *P. vivax* infection [3–8, 12–15]. In many instances, severe thrombocytopaenia was used by the authors of these reports as the stand-alone criterion of malaria severity, irrespective of the presence of haemorrhagic events and/or other signs of disease severity.

However, two relevant aspects deserve to be considered when interpreting the findings of these studies. The first concerns the lack of any specific case definition for severe *P. vivax* malaria and, consequently, the question of whether severe thrombocytopaenia should be considered, in the absence of bleeding, a marker of disease severity.

Thrombocytopaenia (*i.e.*, a platelet count below 150,000/μL) is a frequent haematological finding in all types of malaria and it is observed in 29–93.3 % of patients with *P. vivax* malaria [9, 17]. The exact mechanisms underlying the decrease in platelet counts is still unknown, but various hypothesis have been advanced including immune-mediated phenomena, oxidative stress, alterations in splenic function and a direct interaction between the parasite and platelets [18–22]. Recently, Coelho and coworkers demonstrated that macrophage-driven phagocytosis of platelets may be an important contributory mechanism and that the mean platelet volume was greater in thrombocytopaenic patients with vivax malaria than in controls [23]. The latter finding is particularly interesting because the presence of large circulating platelets and may be viewed as compensatory mechanism in order to preserve primary haemostasis. Accordingly, bleeding is seldomly observed in the course of malaria even among patients with severe thrombocytopaenia. So what is the clinical and prognostic significance of severe thrombocytopaenia in patients with vivax malaria?

Thirty-three case reports of severe thrombocytopaenia (median platelet count: 21,000/μL, range: 2000–45,000/μL) in patients with vivax malaria have been published in the medical literature from 1993 to 2014 (the majority after 2006) [11, 12, 15, 24–51]. Ten individuals presented with haemorrhagic manifestations [27, 28, 30, 35, 38, 40, 41, 43, 48] and nine received platelet transfusions: all patients recovered with the exception of one who died because of shock and pancreatitis [45] (Table 1).

**Table 1:** Published case reports of severe thrombocytopaenia associated with severe *Plasmodium vivax* malaria

Year of publication/reference	Location of malaria acquisition	Age/sex	Platelet count/μL (nadir)	Other complications	Diagnostic method/parasite density	*P. falciparum* excluded	Treatment/platelets transfusion (N unit)	Outcome
1997/[15]	Thailand	27/F	22.000	No	Microscopy/serology/0.6 %	Yes	Sulfadoxine-pyrimethamine + primaquine/no	Recovery
1998/[24]	India	20/M	14.000	Severe anemia (Hb 3 g/dL)	Microscopy/NR	No	Chloroquine + primaquine/no	Recovery
1999/[11]	India	43/F	5.000	No	Microscopy/RDT/NR	Yes	Chloroquine + primaquine/no	Recovery
1999/[11]	Colombia	32/M	17.000	Shock; ARDS	Microscopy/PCR/5 %	Yes	Quinidine + primaquine/no	Recovery
2002/[12]	India	43/M	8.000	No	Microscopy/RDT/NR	Yes	Quinine sulphate/yes (6 units)	Recovery
2003/[26]	India	29/M	35.000	Jaundice	Microscopy/PCR/<1 %	Yes	Chloroquine + primaquine/no	Recovery
2003/[27]	Mexico	30/M	6.000	Epistaxis	Microscopy/NR	No	Quinine + doxycycline switched to chloroquine + primaquine/yes (20 units)	Recovery
2004/[28]	Brazil	20/M	2.000	Petechiae, hemorrhagic bullae	Microscopy/PCR/NR	Yes	Chloroquine/yes (1 unit)/prednisone	Recovery ITP^a^
2005/[29]	Brazil	43/F	17.000	ARDS; shock	Microscopy/RDT/NR	Yes	Chloroquine + artemether + primaquine/No	Recovery
2005/[30]	India	7/M	6.000	Petechial rash, gum bleeding	Microscopy/bone marrow/NR	No	Chloroquine (1 dose) followed by quinine dihydrochloride + primaquine/yes (2 units)/steroids	Recovery
2006/[31]	Venezuela	50/F	25.000	ARDS	Microscopy/PCR/1200/μL	Yes	Mefloquine + primaquine/no	Recovery
2006/[32]	Turkey	22 days/F	17.000	Jaundice	Microscopy/NR	No	Chloroquine + primaquine/yes (NR)	Recovery
2007/[33]	Guyana	59/M	10.000	ARDS; shock; renal failure	Microscopy/PCR/5 %	Yes	Quinine sulphate + doxycycline switched to intravenous quinidine/yes (1 unit)	Recovery
2007/[34]	Pakistan	59/M	39.000	ARDS; renal failure	Microscopy/RDT/PCR/3 %	Yes	Chloroquine + primaquine/no	Recovery
2007/[35]	India	8/M	30.000	Renal failure; petechial rash	Microscopy/RDT/24.000/μL	Yes	Quinine dihydrochloride/no	Recovery
2007/[36]	Republic of Korea	21/M	25.000	Shock	Microscopy/RDT/PCR/2352/μL	Yes	Chloroquine + primaquine/no	Recovery
2007/[36]	Republic of Korea	33/M	40.000	Shock	Microscopy/RDT/12.376/μL	Yes	Chloroquine + primaquine/no	Recovery
2008/[37]	Brazil	14/M	6.000	No	Microscopy/bone marrow/PCR/NR	Yes	Chloroquine + primaquine/no	Recovery
2009/[38]	India	4/F	11.000	Shock; bleeding; increased liver enzymes (AST 1080 U/L)	Microscopy/bone marrow/PCR/RDT/	Yes	Artesunate + mefloquine + primaquine + immunoglobulin/yes	Recovery
2009/[39]	Republic of Korea	27/F	21.000	Myocarditis	Microscopy/PCR/6990/μL	Yes	Chloroquine/no	Recovery
2009/[40]	India	1/M	20.000	Severe anemia; intracranial bleeding; seizures	Microscopy/RDT/NR	No	Cloroquine + quinine dihydrochloride +primaquine/yes	Recovery with hydrocephalus
2010/[41]	Republic of Korea	52/M	40.000	Retinal hemorrhage; jaundice; spleen infarction	Microscopy/9188/μL	No	Chloroquine + primaquine/no	Recovery
2010/[42]	Brazil	16/M	12.000	Rhabdomyolysis, renal failure	Microscopy/PCR/1520/μL	Yes	Chloroquine + artesunate + clindamycin/no	Recovery
2010/[43]	India	8/F	21.000	Petechial rash; severe anemia (Hb 4.7 g/dL); hematemesis	Microscopy/bone marrow/RDT/1.5 %	Yes	Chloroquine + primaquine/no	Recovery
2010/[43]	India	4/F	35.000	Epistaxis; melena	Microscopy/RDT/NR	Yes	Chloroquine + primaquine/yes (NR)	Recovery
2011/[44]	India	50/M	19.000	ARDS, hypotension	Microscopy/NR	No	Artesunate + primaquine/no	Recovery
2012/[45]	India	17/M	34.000	Acute pancreatitis; shock	Microscopy/RDT/NR	Yes	NR/No	Died
2013/[46]	Malaysia	38/M	41.000	ARDS	Microscopy/PCR/16/μL	Yes	Artesunate + primaquine/no	Recovery
2013/[47]	India	19/F	45.000	Acute myocarditis	Microscopy/RDT/NR	Yes	Artesunate + doxycycline/no	Recovery
2013/[48]	India	11/M	27.000	Petechial rash; jaundice; acute renal failure; GI bleeding, shock	Microscopy/RDT/NR	No	Artesunate/yes (NR)	Recovery
2013/[49]	Republic of Korea	59/M	37.000	ARDS; acute renal failure; jaundice; lactic acidosis	Microscopy/RDT/16,380/μL	Yes	Artesunate + chloroquine + primaquine/no	Recovery
2014/[50]	Greece	42/F	24.000	ARDS; jaundice	Microscopy/PCR//0.007 %	Yes	Mefloquine + quinine + doxycycline + primaquine/no	Recovery
2014/[51]	India	8/F	11.000	GCS 7; haemophagocytic syndrome; acute renal failure; hypotension	Microscopy/bone marrow/RDT/NR	Yes	Artesunate + sulphadoxine-pyrimethamine + primaquine/no	Recovery

*F* female, *M* male, *Hb* haemoglobin, *NR* not reported, *RDT* rapid diagnostic tests, *ARDS* acute respiratory distress syndrome, *PCR* polymerase chain reaction, ^a^ *ITP* idiopathic thrombocytopaenic purpura, *AST* alanine aminotransferase, *GCS* Glasgow Coma Score, *GI* gastrointestinal

Information regarding the relationship between vivax malaria and thrombocytopaenia adopted as a criterion for assessing malaria severity may be also extracted from 17 clinical studies (13 from India [13, 14, 52–62], two from Pakistan [63, 64] and one each from Colombia and Sudan [65, 66]). In fifteen of these studies, a cut-off value of less than 50,000 platelets per μL was used to define severe thrombocytopaenia [13, 52–60, 62–66]. Overall, platelet counts below 50,000/μL were present in 335 out of the 906 cases of severe malaria examined (36.9 %), although the prevalence varied significantly among the studies, from 12.5 % to 93 % (Table 2), because of the different selection criteria employed. Bleeding manifestations (mainly epistaxis) were observed in 108 patients (34.8 %), but only in two patients were they serious enough to directly contribute to death (*i.e.*, disseminated intravascular coagulation and gastrointestinal tract haemorrhage) [54, 58].

**Table 2:** Clinical studies using severe thrombocytopaenia as a criterion of severe malaria

References	Type of study/country	No patients with severe *P.vivax* malaria/*P. vivax* malaria (%)	Exclusion *P. falciparum* ^a^/Exclusion of other infectious diseases	No patients with severe thrombocytopaenia among those with severe malaria (%)	No patients with bleeding manifestation (tipology)	Death
[13]	Prospective (adults)/India	40/456 (0.08)	Yes (RDT)/PCR/Yes	5 (12.5)	2 (Severe epistaxis requiring blood and platelet transfusions)	2 due to ARDS (none with Thp)
[14]	Prospective (pediatrics)/India	24/35 (68.6)	No/No	17 (70.8)^b^	2 (Epistaxis)	None
[52]	Retrospective (adults and pediatrics)/India	17/221 (7.7)	No/No	13	NR	3 (Pregnant women with ARDS)
[53]	Retrospective (adults)/India	28/30 (93.3)	No/No	28 (93.3)	None	2:1 ARDS, 1 CM (none with Thp)
[54]	Retrospective (pediatrics)/India	23/108 (21.3)	Yes (RDT)/No	9/23 (39.1)	3 (Petechiae, purpura)	1: GI bleeding, DIC, renal failure
[55]	Retrospective (adults)/India	43/121 (35.5)	Yes (RDT)^c^/No	43	None	3 due to ARDS (none with Thp)
[56]	Prospective (pediatrics)/India	60/380 (15.8)	Yes (RDT)/Yes	51/60 (85)	44 (Epistaxis); 19 (hematemesis)	None
[57]	Retrospective (adults)/India	107/213 (50.2)	Yes (RDT)/No	17 (15.8)	None	None
[58]	Retrospective (pediatrics)/India	45/131 (21.1)	Yes (RDT)/No	17 (13)	12 (NR)	4:2 ARDS/ARF; 1 CM; 1 DIC
[59]	Retrospective (pediatrics)/India	54/261 (20.7)	Yes (RDT)/Yes	20 (37)	10 (NR)	None
[60]	Prospective (adults and pediatrics)/India	200/900 (22.2)	Yes (RDT)/No	24/200 (12)	4 (Purpuric eruptions)	40
[61]	Prospective (adults)/India	22/198 (11.1)	Yes (RDT + PCR)/No	7/14 (50)^d^	9 (NR)	2 CM and ARDS (none with Thp)
[62]	Prospective (pediatrics)/India	38/61 (62.3)	Yes (RDT)/No	18/38 (47.4)	NR	5 (3 ARDS)
[63]	Prospective (pediatrics)/Pakistan	397,128 (30.5)	No/No	15 (38.5)	NR	1 with GCS <10, convulsion (without Thp)
[64]	Retrospective (adults)/India	111/296 (37.5)	Yes (RDT + PCR)^e^/No	58/111 (52.2)	16 (GI, genitourinary or respiratory tracts)	3 8 acute myocardial infarction)
[65]	Retrospective (adults and pediatrics)/India	83/359 (23.1)	No/No	13 (15.7)	4 (NR)	None
[66]	Prospective (pediatrics)/Sudan	18/18 (100)	No/No	4 (22.2)	2 epistaxis	None
Total		952/3916 (24.3)	11/17 (64.7)/3/17 (17.6)	359/952 (37.7)	106/3506 (3)/106/310 (34.2)	66/952 (6.9)

^a^By polymerase chain reaction (PCR) or rapid diagnostic tests (RDTs)

^b^All patients with less than 150.000 per μL were considered

^c^Only some patients underwent RDT analysis

^d^In this study severe thrombocytopaenia was considered with value <80.000 per μL

^e^Only discordant samples

*RDT* rapid diagnostic test, *PCR* polymerase chain reaction, *NR* not reported, *Thp* thrombocytopaenia, *CM* cerebral malaria, *ARDS* acute respiratory distress syndrome

It is noteworthy that mixed *P. vivax* and *P. falciparum* infections were not ruled out in 14 out of 17 studies [14, 52, 53, 63–66]. In addition, coexisting infections that may contribute to thrombocytopaenia (such as dengue fever, leptospirosis or bacterial sepsis) were not excluded in the majority of the studies [14, 52–55, 57, 58, 60–66]. Regarding the latter issue, a recent study conducted in the Brazilian Amazon showed that 17.6 % of patients with *P. vivax* malaria had concomitant dengue infection and these had a higher probability to present with haemorrhagic manifestations and jaundice [67].

Other studies have focused on the prognostic role of thrombocytopaenia in vivax malaria. Leal-Santos and coworkers in a cross-sectional study conducted in 186 patients from the Mato Grosso region showed that mean platelet volume and platelet distribution width (PDW) were significantly associated with the presence of warning signs of severe and complicated malaria (*i.e.*, anaemia, hypotension and elevated creatinine levels) with odds ratios (OR) of 3.47 and 5.44, respectively [68]. In keeping with the above mentioned results, a large study conducted in Papua New Guinea showed that the greatest risk of severe thrombocytopaenia was associated with *P. falciparum* malaria (OR 6.03 vs 3.73 for *P. vivax*). However, the mortality risk for patients with severe thrombocytopaenia was higher among patients without malaria (7.9 %) than among those with *P. falciparum* (2.1 %) or *P. vivax* (1.5 %) malaria [69]. The authors also found a 16-fold higher risk of death when severe thrombocytopaenia was associated with severe anaemia (haemoglobin concentration below 5 mg/dL), and concluded that severe thrombocytopaenia should serve as a warning sign of poor outcome in patients with malaria particularly when it is accompanied by severe anaemia. Based on their findings, Lampah and coworkers proposed a threshold of ≤20,000 platelets/μL as a defining severity criterion for both *P. falciparum* and *P. vivax* malaria [69].

However, in a recent study conducted in Brazil and India on 778 patients with documented *P. vivax* monoinfection, platelet counts showed a very poor discriminative performance to identify criteria of severe disease and the authors concluded that thrombocytopaenia should not be used to identify patients with *P. vivax* complications [70].

The second issue that deserves to be addressed when considering disease severity regards mortality. In other words: did patients whose death was attributed to *P. vivax* malaria die of malaria or with malaria?

This issue has been addressed in one study only which was conducted in Brazil and reported the autopsy findings of 17 patients who died of *P. vivax* malaria [5]. The authors reported during the study period (1996–2010) a case-fatality rate (CFR) for *P. vivax* malaria of 0.011 % (19/170.286), but in only four of the seventeen patients who underwent autopsy death could be exclusively attributed to *P. vivax* malaria, resulting in a CFR of 0.002 % [5]. Of note, the CFR of *P. vivax* malaria was 16-fold lower than the one observed for *P. falciparum* malaria in the same period (0.032 %, 12/36.854) [5]. In another study conducted in Papua, Indonesia, between January 2004 and September 2009, a post hoc clinical death audit of cases of pure *P. vivax* malaria resulted in a CFR of 0.12 per 1000 infections [71]. Of note, of the six fatal cases for whom vivax malaria was considered the primary cause of death, four were young children of less than 2 years of age (two of whom were malnourished), and the other two were adults in whom sepsis was concomitantly documented.

Moreover, in a retrospective analysis of 12.769 cases of imported *P. vivax* malaria observed in the UK over 27 years (1987–2013), seven deaths were registered, giving an overall mortality of 0.05 %. The median age of deceased patients was 72 years, while no deaths occurred in the 9927 patients aged less than 50 years [72]. In the two studies regarding imported *P. vivax* malaria conducted by the TropNetEurope and the GeoSentinel network, reporting respectively 618 and 278 patients (either travellers or immigrants), no deaths were recorded among patients affected by this species of *Plasmodium* [73, 74].

Thus, based on available data, the mortality risk of *P. vivax* malaria seems to be different in endemic areas as opposed to what is observed in cases of imported disease in Europe or USA. In endemic areas, the risk of death is low and influenced by several factors, such as very young age, severe anaemia at presentation, malnutrition, pregnancy, coexisting comorbidities and concurrent infections [69–71, 75]. When imported *P. vivax* malaria is considered, the risk of death is generally absent or negligible and principally influenced by old age. Another important issue that should be considered when comparing malaria mortality is the lack of high level supportive care in many countries where malaria is endemic a factor that explains the better outcome observed in non-endemic countries where high-quality healthcare is available [76]. Despite these considerations it should be emphasized that the risk of death directly attributable to *P. vivax* malaria should not be overlooked.

In conclusion, given the lack of high quality studies and the fact that several confounding factors cannot be ruled out in the majority of case reports and studies published so far, the role of severe thrombocytopaenia as an indicator of *P. vivax* malaria severity cannot at present neither be discarded nor confirmed and it deserves to be addressed in well conducted prospective studies in both endemic and non-endemic countries.

## Abbreviations

ED: emergency department
WHO: World Health Organization
ICU: intensive care unit
G6PD: glucose-6-phosphate dehydrogenase
PCR: polymerase chain reaction
CFR: case-fatality rate
DIC: disseminated intravascular coagulation
